# Effect of Sub-antagonist Doses of Naloxone as an Adjuvant to Ropivacaine on the Onset and Quality of Axillary Brachial Plexus Block in Patients Undergoing Forearm and Hand Surgery: A Randomized Double-Blind Clinical Trial

**DOI:** 10.5812/aapm-172772

**Published:** 2026-06-30

**Authors:** Behrooz Zaman, Masood Mohseni, Seyed Alireza Seyed Siamdoust, Nasrin Nouri, Samad Noorizad, Taymaz Amiraslani, Mahshad Ghezelbash

**Affiliations:** 1 Department of Anesthesiology and Pain Medicine, Pain Research Center, School of Medicine, Iran University of Medical Sciences Tehran Iran; 2 Department of Anesthesiology and Pain Medicine, School of Medicine, Iran University of Medical Sciences Tehran Iran

**Keywords:** Axillary Brachial Plexus Block, Naloxone, Ropivacaine, Regional Anesthesia, Sub-antagonist Dose, Hand and Forearm Surgery, Adjuvant, Peripheral Nerve Block

## Abstract

**Background:**

Axillary brachial plexus block (ABPB) is the preferred regional anesthetic technique for forearm and hand surgery. Naloxone at sub-antagonist doses may paradoxically enhance local anesthetic effects through selective opioid receptor modulation; however, the optimal dose and its clinical benefit in ABPB with ropivacaine remain unclear.

**Objectives:**

This study aimed to compare the effects of ultra-low-dose naloxone (0.4 µg), low-dose naloxone (4 µg), and placebo added to 0.5% ropivacaine on block onset, intensity, and postoperative analgesia in patients undergoing elective forearm and hand surgery. To our knowledge, this is the first trial to directly compare two sub-antagonist doses of naloxone as adjuvants to ropivacaine for axillary block.

**Methods:**

In this randomized, double-blind clinical trial, 120 patients with American Society of Anesthesiologists (ASA) physical status I or II were allocated equally to three groups: Group 1 (control; ropivacaine plus saline), Group 2 (ropivacaine plus low-dose naloxone, 4 µg), and Group 3 (ropivacaine plus ultra-low-dose naloxone, 0.4 µg). All blocks were performed using combined ultrasound and nerve stimulator guidance. The co-primary outcomes were sensory and motor block onset times. Secondary outcomes included block intensity, visual analog scale (VAS) pain scores during recovery, and the proportion of patients requiring pethidine within 24 hours.

**Results:**

All 120 blocks (100%) were clinically successful, with no intraoperative rescue analgesia or conversion to general anesthesia in any group. Motor block onset was significantly faster in Groups 2 and 3 than in Group 1 (P < 0.001); no patient in the naloxone groups required 9 minutes to achieve motor block, compared with 30% in Group 1. Sensory block onset did not differ significantly among groups (P = 0.163). At 30 minutes, VAS scores were lower in both naloxone groups than in Group 1; the overall analysis of variance (ANOVA) result was P < 0.001, and the reported comparison between Group 3 and Group 1 was significant (P < 0.001). The 15-minute difference was not significant after *Bonferroni correction*. The proportion requiring pethidine within 24 hours was significantly lower in Group 3 than in Group 1, with a risk ratio of 0.52 [95% CI, 0.33 - 0.82] (27.5% vs. 52.5%; P = 0.005). Group 2 had a numerically higher pethidine requirement than Group 1 (62.5% vs. 52.5%), suggesting possible dose-dependent partial receptor antagonism that requires confirmation in future studies.

**Conclusions:**

Ultra-low-dose naloxone (0.4 µg) significantly accelerates motor block onset and reduces postoperative pethidine requirements in patients with American Society of Anesthesiologists physical status I or II undergoing elective hand and forearm surgery with ABPB, without adverse effects. Although the 4 µg dose was associated with lower VAS scores at 30 minutes, it did not reduce pethidine requirements and was associated with a nonsignificant increase in the proportion of patients requiring pethidine, consistent with a biphasic dose-response model. These findings highlight the narrow therapeutic margin of perineural naloxone and suggest caution with doses above the ultra-low-dose range, as higher sub-antagonist doses may paradoxically antagonize endogenous analgesic mechanisms.

## 1. Background

Regional anesthesia has become the preferred technique for upper extremity surgery, providing superior postoperative analgesia, reduced systemic opioid exposure, faster recovery, and higher patient satisfaction compared with general anesthesia (1-3). Axillary brachial plexus block (ABPB) is among the most commonly used peripheral nerve block approaches for procedures distal to the elbow. Its favorable safety profile, notably the absence of pneumothorax, phrenic nerve palsy, and Horner's syndrome associated with more proximal approaches, makes it the regional technique of choice for forearm and hand surgery (4).

Despite these advantages, a clinically relevant limitation of single-injection ABPB is its moderate onset time and finite duration of analgesia. Optimizing block quality remains an active area of investigation, as incomplete nerve coverage can result in tourniquet pain despite adequate surgical anesthesia (5). A range of adjuvants has been evaluated to enhance block onset and quality, including opioids, clonidine, dexmedetomidine, dexamethasone, magnesium sulfate, and ketamine (6, 7). In axillary blocks for hand and forearm surgery, Zaman et al. demonstrated that combined ketamine and dexamethasone adjuvants significantly affected block onset and duration, supporting pharmacological adjuvant strategies in this setting (8). However, not all adjuvants are effective. Hemati et al. showed that neostigmine added to 0.5% ropivacaine for an axillary block provided no significant analgesic benefit, underscoring the need for rigorous, dose-specific evaluation before clinical adoption (9). Among available agents, dexmedetomidine and dexamethasone have the strongest evidence for prolonging peripheral nerve block duration (10, 11). A 2021 network meta-analysis by Sehmbi et al. demonstrated that both perineural dexamethasone and dexmedetomidine significantly prolonged sensory and motor block in supraclavicular brachial plexus block (12). However, dexmedetomidine carries risks of bradycardia and hypotension, whereas dexamethasone raises concerns regarding systemic steroid effects, prompting continued interest in alternative adjuvants with more favorable safety profiles (13).

Systematic reviews have confirmed growing interest in identifying safe and effective adjuvants for peripheral nerve blocks. Chong et al. (14) demonstrated that perineural dexamethasone provides superior analgesia compared with intravenous administration, whereas Vorobeichik et al. (15) confirmed the efficacy of dexmedetomidine in brachial plexus blocks. Both agents have specific safety concerns that have prompted investigation of alternative adjuvants.

Naloxone, a competitive opioid receptor antagonist, has attracted attention as a local anesthetic adjuvant at sub-antagonist concentrations. Paradoxically, at nanomolar to picomolar doses, naloxone may enhance rather than antagonize analgesia. As originally demonstrated by Crain and Shen, ultra-low-dose opioid antagonists selectively block Gs-coupled (excitatory) opioid receptor functions while preserving or potentiating Gi-coupled (inhibitory) analgesic signaling, a mechanism fundamentally different from conventional opioid antagonism (16, 17). This bidirectional receptor modulation may augment the analgesic effects of local anesthetics through increased endogenous opioid release and enhanced peripheral opioid receptor sensitivity.

Subsequent preclinical studies have further explored the mechanistic basis of this phenomenon. Guan et al. demonstrated that peripherally acting mu-opioid receptor agonists attenuate neuropathic pain in animal models, supporting peripheral opioid receptor modulation as a viable analgesic strategy (18). Other work has suggested that ultra-low-dose naloxone may also modulate Toll-like receptor 4 signaling and reduce neuroinflammation, although these mechanisms remain speculative (19).

Movafegh et al. were among the first to demonstrate clinical applicability, reporting that ultra-low-dose naloxone added to lidocaine or a lidocaine-fentanyl mixture significantly prolonged ABPB duration (20). In supraclavicular brachial plexus block, Marashi et al. reported prolonged sensory and motor blockade with naloxone (21), whereas Al-Shukaili et al. reported prolonged sensory block but shortened motor block (22). Lee et al. confirmed that low-dose naloxone added to ropivacaine prolonged femoral nerve block and reduced 24-hour opioid consumption (23). The safety of peripheral nerve block adjuvants is a critical clinical consideration, as highlighted by El-Boghdadly et al., who reviewed local anesthetic systemic toxicity and emphasized careful adjuvant selection and dosing (24).

## 2. Objectives

Despite these encouraging findings, the optimal sub-antagonist dose of naloxone as an adjuvant for peripheral nerve block remains undefined. To date, no trial has directly compared multiple sub-antagonist doses of naloxone in ABPB with ropivacaine. Therefore, the present study was designed to compare ultra-low-dose naloxone (0.4 µg), low-dose naloxone (4 µg), and placebo combined with 0.5% ropivacaine with respect to ABPB onset, intensity, and postoperative analgesia in patients undergoing forearm and hand surgery.

## 3. Methods

### 3.1. Study Design and Ethics

This prospective, randomized, double-blind clinical trial was conducted at Hazrate-Fatemeh Hospital, Iran University of Medical Sciences, Tehran, Iran, between May 2022 and October 2023, in accordance with the Declaration of Helsinki. The study was reported in accordance with the CONSORT 2010 guidelines. The protocol was approved by the Ethics Committee of the Faculty of Medicine, Iran University of Medical Sciences (ethics code: IR.IUMS.FMD.REC.1401.498), and registered in the Iranian Registry of Clinical Trials (IRCT20170105031787N6). Written informed consent was obtained from all participants before enrollment.

### 3.2. Participants

Patients aged 18 - 70 years with American Society of Anesthesiologists (ASA) physical status I or II who were scheduled for elective forearm and hand surgery under ABPB were eligible. The exclusion criteria were a known allergy to local anesthetics or naloxone, congenital or acquired neuromuscular disorders, a history of seizures, opioid dependence, cardiac disease, gastrointestinal disorders, and refusal to provide written consent.

### 3.3. Randomization and Blinding

Eligible patients were randomly allocated to three equal groups (n = 40 each) using block randomization performed by an independent statistician. Patients were informed that they would be randomly assigned to one of three treatment groups but were not informed of their specific allocation. Study solutions were prepared by a nurse anesthetist who was not involved in patient care or outcome assessment. All syringes were identical in appearance and labeled only with coded numbers (Groups 1, 2, and 3), without drug names.

This was a double-blind study: both patients and the outcome assessor (the anesthesiologist performing postoperative evaluations) were masked to group allocation. The surgeons, data collector, and data analyst were also blinded to treatment assignments.

The anesthesiologist administering the block was aware of the solution contents but was excluded from all postintervention evaluations and data handling. All outcomes were assessed by a separate anesthesiologist who was not present during the block procedure. This approach maintained blinding while ensuring patient safety during the invasive procedure.

### 3.4. Interventions

Standard monitoring, including electrocardiography, SpO_2_, and noninvasive blood pressure measurement, was applied, and intravenous access was established in all patients. Preblock sedation consisted of intravenous midazolam 0.15 mg/kg and fentanyl 1.5 µg/kg, administered uniformly to all groups 10 minutes before block performance. This standardized approach ensured that between-group differences in outcomes could be attributed to the study intervention rather than to variations in premedication. The block was performed under simultaneous real-time ultrasound guidance with a high-frequency linear-array probe (6 - 13 MHz) and nerve stimulator confirmation. A 22-gauge, 50-mm insulated stimulating needle (Stimuplex® A, *B. Braun*, Germany) was used for all blocks. Ultrasound guidance was performed using a SonoSite M-Turbo device, and nerve stimulation was performed using a Stimuplex HNS 12 device (*B. Braun*, Germany).

The four main brachial plexus nerves (median, ulnar, radial, and musculocutaneous) each received 7 mL of the study solution (total, 28 mL). The intercostobrachial nerve, a separate cutaneous nerve that does not arise from the brachial plexus, was also targeted under ultrasound guidance with 2 mL of the study solution to provide coverage for tourniquet pain. Thus, the total volume of 30 mL per patient comprised 28 mL for the four brachial plexus nerves and 2 mL for the intercostobrachial nerve.

The study solutions were prepared as follows:

Group 1 (control): 29 mL of 0.5% ropivacaine (Varian Pharmamed) plus 1 mL of normal saline (total volume, 30 mL).

Group 2 (low-dose naloxone): 29 mL of 0.5% ropivacaine (Varian Pharmamed) plus 1 mL containing naloxone 4 µg (Tolid Daroo) (total volume, 30 mL).

Group 3 (ultra-low-dose naloxone): 29 mL of 0.5% ropivacaine (Varian Pharmamed) plus 1 mL containing naloxone 0.4 µg (Tolid Daroo) (total volume, 30 mL).

All syringes were identical in appearance and labeled only with codes to maintain blinding.

### 3.5. Outcome Measures

The co-primary outcomes were sensory and motor block onset times, as specified in the original protocol and sample size calculation. Because these outcomes are correlated components of peripheral nerve block assessment, no formal multiplicity adjustment was applied to the two co-primary endpoints. A union-intersection approach was used to determine overall onset success: Success was defined as a statistically significant between-group difference in at least one co-primary outcome (sensory or motor block onset). This approach was selected because sensory and motor onset represent complementary but distinct aspects of block performance. Requiring both to be significant, using an intersection-union approach, would be overly conservative and would substantially increase the risk of a type II error. Secondary outcomes were block intensity, evaluated by intraoperative rescue analgesic requirements; visual analog scale (VAS) pain scores, ranging from 0 to 10, at 5, 15, 30, and 60 minutes during recovery; and the proportion of patients requiring pethidine within 24 hours. Block duration was not formally assessed as a prespecified outcome because of limitations in the original data collection protocol; this is acknowledged as a study limitation.

Sensory block was assessed using the pinprick test every 3 minutes, and motor block was assessed using finger flexion and extension every 3 minutes. If sensory or motor block had not been achieved at an assessment, its absence was recorded, and assessment continued at the next scheduled interval. If VAS ≥ 3 during surgery, intravenous fentanyl 1 µg/kg was administered as rescue analgesia; patients requiring a second rescue dose were converted to general anesthesia. Systolic blood pressure (SBP), diastolic blood pressure (DBP), and heart rate (HR) were monitored throughout. A fixed dose of pethidine 50 mg was administered if VAS ≥ 3 during recovery.

### 3.6. Statistical Analysis

The sample size was calculated based on Lee et al. (23), with α = 0.05 and 80% power, yielding 40 patients per group (total n = 120). All analyses were performed on an intention-to-treat basis. All 120 randomized patients completed the study without loss to follow-up or protocol deviations. Continuous variables are presented as mean ± standard deviation (SD), and categorical variables as frequencies and percentages. Between-group comparisons were performed using one-way analysis of variance (ANOVA) with Tukey's post hoc test for continuous variables and the chi-square or Fisher's exact test for categorical variables. For exploratory VAS comparisons at four time points, *Bonferroni correction* was applied, with statistical significance set at P < 0.0125 (0.05/4). No formal multiplicity adjustment was applied to the two co-primary outcomes, as they are inherently correlated components of the same physiological process (peripheral nerve block onset) and were prespecified in the original protocol and sample size calculation. Consistent with the union-intersection approach described in Section 3.5, overall onset success was defined as a statistically significant between-group difference in at least one co-primary outcome. This approach controls the family-wise error rate while preserving statistical power and is appropriate when co-primary endpoints are correlated and measure complementary dimensions of the same clinical phenomenon. Findings with P values between 0.0125 and 0.05 were interpreted with caution. Normality of continuous variables was assessed using the Kolmogorov-Smirnov test. The VAS scores had a nonnormal distribution with substantial zero inflation; therefore, the Kruskal-Wallis test with Dunn's post hoc correction was also performed as a sensitivity analysis. These results were consistent with the primary ANOVA findings. Effect sizes are reported as mean differences with 95% confidence intervals for continuous outcomes and risk ratios with 95% confidence intervals for binary outcomes. A two-tailed P < 0.05 was considered statistically significant for primary outcomes. All analyses were performed using SPSS version 25 (IBM Corp., Armonk, NY, USA).

## 4. Results

### 4.1. Participant Flow and Baseline Characteristics

Of 128 patients assessed for eligibility, 8 were excluded: 1 due to a history of cardiac disease, 2 due to an allergy to local anesthetics, and 5 because they declined consent. A total of 120 patients were enrolled and randomized into three equal groups (n = 40 each) (Figure 1). All 120 patients completed the study, with no loss to follow-up or discontinuation.

**Figure 1.**
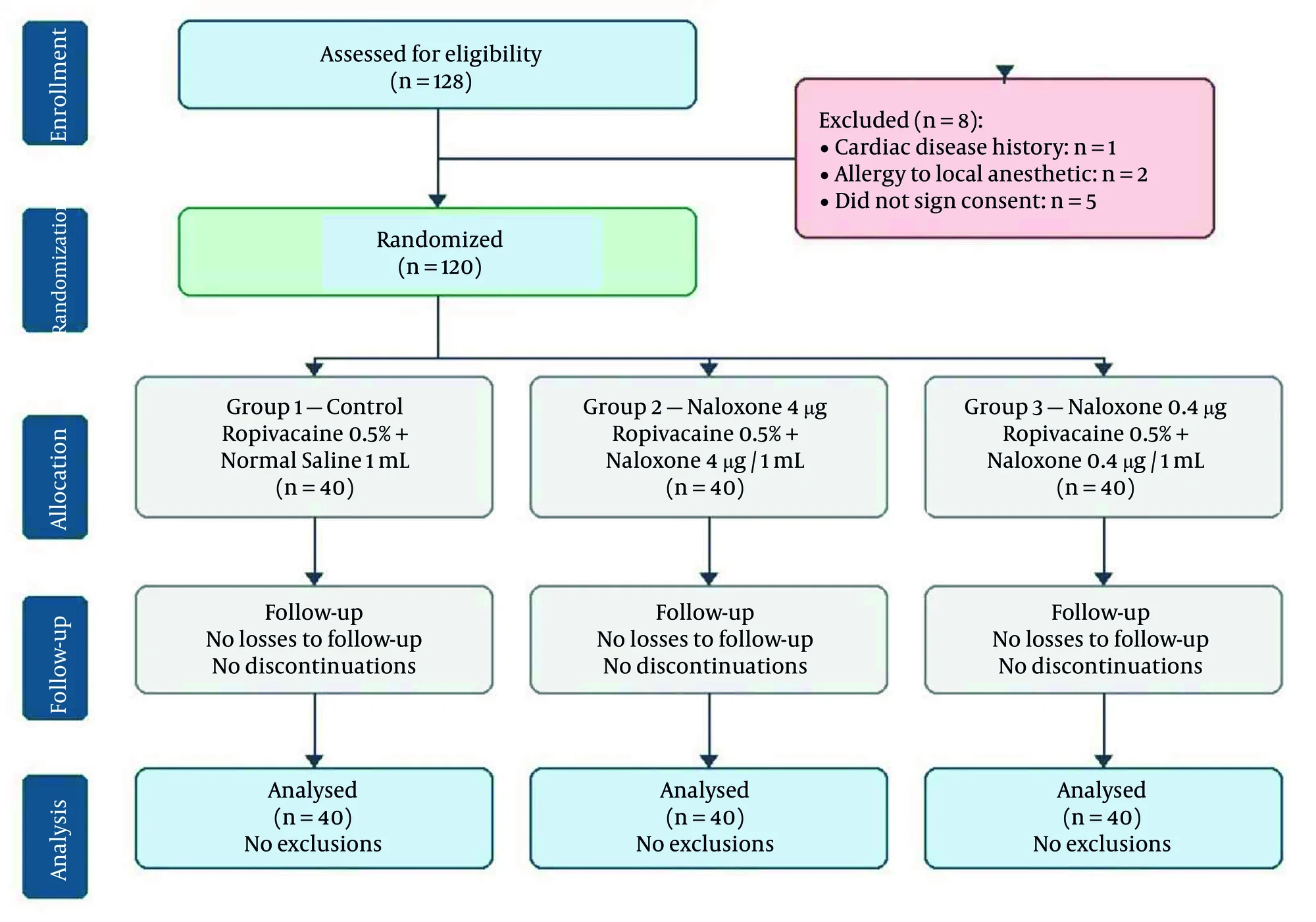
CONSORT 2010 flow diagram. Of 128 patients assessed, 8 were excluded (cardiac disease, n = 1; allergy to local anesthetics, n = 2; declined consent, n = 5). A total of 120 patients were randomized to three equal groups (n = 40 each); all completed the study without loss to follow-up.

The cohort comprised 100 male patients (83.3%) and 20 female patients (16.7%), with a mean age of 41.72 ± 16.68 years. Age, sex, body mass index (BMI), and ASA classification were comparable among groups (P > 0.05 for all). Baseline DBP differed significantly among groups (Group 1, 87.80 ± 5.86; Group 2, 86.43 ± 4.37; Group 3, 83.75 ± 5.88 mm Hg; P = 0.004), indicating a potential baseline imbalance despite randomization. Baseline SBP did not differ significantly among groups (P = 0.546). Demographic data are presented in Table 1.

**Table 1.** Demographic and Baseline Characteristics of the Study Groups (n = 40) ^a^

Variables	Group 1 (Control)	Group 2 (Naloxone 4 µg)	Group 3 (Naloxone 0.4 µg)	P-Value
**Age (y)**	46.38 ± 20.43	38.88 ± 12.45	39.90 ± 15.59	0.091
**Gender**				0.398
Male	35 (87.5)	31 (77.5)	34 (85.0)	
Female	5 (12.5)	9 (22.5)	6 (15.0)	
**BMI (kg/m^2^)**	25.42 ± 2.75	26.36 ± 4.20	24.45 ± 4.02	0.064
**ASA**				0.095
I	30 (75)	36 (90)	36 (90)	
II	10 (25)	4 (10)	4 (10)	
**Baseline SBP (mm Hg)**	127.53 ± 8.83	127.20 ± 6.84	125.80 ± 7.92	0.546
**Baseline DBP (mm Hg) ^b^**	87.80 ± 5.86	86.43 ± 4.37	83.75 ± 5.88	0.004 ^c^

^a^ Values are expressed as mean ± SD or No. (%). Abbreviations: BMI, Body Mass Index; SBP, systolic blood pressure; DBP, diastolic blood pressure; ASA, American Society of Anesthesiologists; SD, standard deviation.

^b^ Acknowledged as a potential baseline imbalance despite randomization. Effect size for the DBP difference between Groups 1 and 3: mean difference, 4.05 mm Hg [95% CI, 1.55 - 6.55]; Cohen's d = 0.69.

^c^ Statistically significant. Tukey post hoc comparison: Group 1 vs. Group 3, P = 0.001.

### 4.2. Sensory Block Onset

Sensory block was achieved at 3 minutes in 70.0%, 77.5%, and 87.5% of patients in Groups 1, 2, and 3, respectively. The remaining patients achieved sensory block at 6 minutes (30.0%, 22.5%, and 12.5%, respectively). Although the trend favored Group 3, the overall difference was not statistically significant (chi-square = 3.633; P = 0.163). Data are shown in Table 2.

**Table 2.** Sensory Block Onset Distribution Across Groups (n = 40) ^a^

Time to Sensory Block (min)	Group 1 (Control)	Group 2 (Naloxone 4 µg)	Group 3 (Naloxone 0.4 µg)	P Value
**3 min**	28 (70.0)	31 (77.5)	35 (87.5)	0.163 ^b^
**6 min**	12 (30.0)	9 (22.5)	5 (12.5)	

^a^ Values are expressed as No. (%).

^b^ The P value represents the overall chi-square test across all time points (chi-square = 3.633; P = 0.163). No statistically significant difference was observed among groups. Effect size: Cramer's V = 0.174.

### 4.3. Motor Block Onset

Motor block onset differed significantly among groups (chi-square = 27.39; P < 0.001). In Group 1, 22.5% achieved motor block at 3 minutes, 47.5% at 6 minutes, and 30.0% required 9 minutes. In Groups 2 and 3, no patient required 9 minutes; motor block was achieved at 6 minutes in 77.5% and 75.0%, respectively. Post hoc analysis confirmed significant differences between Group 1 and both naloxone groups; Groups 2 and 3 did not differ significantly. The effect size for motor block acceleration was large (Cramer's V = 0.338). Data are shown in Table 3.

**Table 3.** Motor Block Onset Distribution Across Groups (n = 40) ^a^

Time to Motor Block (min)	Group 1 (Control)	Group 2 (Naloxone 4 µg)	Group 3 (Naloxone 0.4 µg)	P Value
**3 min**	9 (22.5)	9 (22.5)	10 (25.0)	< 0.001 ^b^^, ^^c^
**6 min**	19 (47.5)	31 (77.5)	30 (75.0)	
**9 min**	12 (30.0)	0 (0.0)	0 (0.0)	

^a^ Values are expressed as No. (%).

^b^ Statistically significant.

^c^ The P value represents the overall chi-square test across all time points (chi-square = 27.39; P < 0.001). Post hoc comparisons: Group 1 vs. Group 2, P < 0.001; Group 1 vs. Group 3, P < 0.001; Group 2 vs. Group 3, P = 1.000. Effect size: Cramer's V = 0.338.

### 4.4. Block Intensity

All 120 blocks were clinically successful across the three groups. No patient required intraoperative fentanyl rescue analgesia or conversion to general anesthesia due to an inadequate block. This 100% success rate confirms that neither ultra-low-dose nor low-dose naloxone compromised the fundamental anesthetic efficacy of 0.5% ropivacaine. Block intensity was adequate for surgical anesthesia in all groups.

### 4.5. Postoperative Pain Scores

VAS scores at 15 and 30 minutes were lower in Group 3 than in Group 1 (Tukey P = 0.031 and P < 0.001, respectively). At 5 minutes, the overall ANOVA result was P = 0.056; the Tukey post hoc comparison between Groups 1 and 3 yielded P = 0.045. After *Bonferroni* correction for four VAS time points (adjusted significance threshold, P < 0.0125), neither the 15-minute overall ANOVA result (P = 0.032) nor the Group 1–Group 3 Tukey comparison (P = 0.031) met the corrected threshold. The 30-minute overall ANOVA result and the reported Group 1–Group 3 comparison remained significant (P < 0.001). At 30 minutes, Group 2 also had a lower mean VAS score than Group 1, with the same reported mean difference and confidence interval as Group 3 (Table 4). No significant overall difference was observed at 60 minutes (P = 0.119). Group 2 did not differ significantly from Group 1 at 5 or 15 minutes. The clinical significance of the Group 3 pain findings is supported by the reduced proportion requiring pethidine within 24 hours. Effect sizes for VAS comparisons are reported as mean differences with 95% confidence intervals in Table 4.

**Table 4.** Visual Analog Scale Pain Scores During Recovery ^a^

Time Point (min)	Group 1 (Control)	Group 2 (Naloxone 4 µg)	Group 3 (Naloxone 0.4 µg)	Comparison	Mean Difference (95% CI)	Cohen's d	Overall ANOVA P Value
**5 min**	0.25 ± 0.67	0.10 ± 0.44	0.00 ± 0.00	G1 – G2	0.15 (-0.102 to 0.402)	0.265	0.056 ^b^
				G1 – G3	0.25 (0.039 to 0.461)	0.528	
				G2 – G3	0.10 (-0.039 to 0.239)	0.321	
**15 min**	0.40 ± 1.13	0.10 ± 0.44	0.00 ± 0.00	G1 – G2	0.30 (-0.082 to 0.682)	0.350	0.032 ^c^
				G1 – G3	0.40 (0.044 to 0.756)	0.501	
				G2 – G3	0.10 (-0.039 to 0.239)	0.321	
**30 min**	0.40 ± 0.81	0.00 ± 0.00	0.00 ± 0.00	G1 – G2	0.40 (0.145 to 0.655)	0.698	< 0.001 ^c^
				G1 – G3	0.40 (0.145 to 0.655)	0.698	
				G2 – G3	0.00 (0.000 to 0.000)	0.000	
**60 min**	0.00 ± 0.00	0.20 ± 0.61	0.20 ± 0.61	G1 – G2	-0.20 (-0.392 to -0.008)	-0.464	0.119
				G1 – G3	-0.20 (-0.392 to -0.008)	-0.464	
				G2 – G3	0.00 (-0.272 to 0.272)	0.000	

^a^ Values are expressed as mean ± SD unless otherwise indicated. The P values in the final column are overall ANOVA results for each time point; they are not pairwise P values. Group means and overall P values apply to all three comparison rows at the corresponding time point. Abbreviations: VAS, visual analog scale, range 0 - 10; SD, standard deviation; CI, confidence interval; ANOVA, analysis of variance; G1, Group 1; G2, Group 2; G3, Group 3.

^b^ At 5 minutes, overall ANOVA P = 0.056; the Tukey post hoc Group 1–Group 3 comparison yielded P = 0.045.

^c^ At 15 minutes, overall ANOVA P = 0.032 and the Group 1–Group 3 Tukey P = 0.031; neither meets the *Bonferroni*-adjusted threshold of P < 0.0125 for four time points. At 30 minutes, overall ANOVA and the reported Group 1–Group 3 Tukey comparison yielded P < 0.001. Low mean VAS values reflect the high block success rate; individual scores ranged from 0 to 4.

### 4.6. Pethidine Requirement Within 24 Hours

Within 24 hours, 52.5% of patients in Group 1 required pethidine 50 mg, compared with 62.5% in Group 2 and 27.5% in Group 3. The overall between-group difference was statistically significant (chi-square = 10.43; P = 0.005). Post hoc analysis showed a significantly lower proportion requiring pethidine in Group 3 than in Group 1, with a risk ratio of 0.52 [95% CI, 0.33 - 0.82] (P = 0.005). The difference between Groups 2 and 3 was also significant, with a risk ratio of 2.27 [95% CI, 1.31 - 3.94] (P = 0.003). No significant difference was found between Groups 1 and 2 (P = 0.334). Group 2 had a numerically higher pethidine requirement than Group 1 (62.5% vs. 52.5%), although the difference was not statistically significant. Data are presented in Table 5.

**Table 5.** Pethidine Requirement Within 24 Hours Postoperatively (n = 40) ^a^

Pethidine Requirement	Group 1 (Control)	Group 2 (Naloxone 4 µg)	Group 3 (Naloxone 0.4 µg)	Overall P Value	Risk Ratio [95 CI]
**No pethidine required**	19 (47.5)	15 (37.5)	29 (72.5)	0.005 ^b^^, ^^c^	Group 3 vs. Group 1: 0.52 [0.33 - 0.82]
**Pethidine 50 mg required**	21 (52.5)	25 (62.5)	11 (27.5)		Group 2 vs. Group 1: 1.19 [0.83 - 1.71]
					Group 2 vs. Group 3: 2.27 [1.31 - 3.94]

^a^ Values are expressed as No. (%). Abbreviation: CI, confidence interval.

^b^ Statistically significant.

^c^ The P value represents the overall chi-square test (chi-square = 10.43; P = 0.005). Post hoc comparisons: Group 1 vs. Group 3, P = 0.005; Group 1 vs. Group 2, P = 0.334; Group 2 vs. Group 3, P = 0.003. Risk ratios refer to requiring pethidine within 24 hours, regardless of the display row.

### 4.7. Hemodynamic Parameters

Hemodynamic parameters (SBP, DBP, and HR) remained stable throughout the intraoperative period and recovery room stay, with no clinically significant differences among groups.

### 4.8. Safety

All 120 blocks were performed successfully, with no block failures. One patient in Group 1 experienced mild nausea in the recovery room. No local anesthetic systemic toxicity, neurological deficits, hematoma, or other adverse events attributable to the study interventions were recorded in any group.

## 5. Discussion

This randomized trial demonstrates three principal findings. First, ultra-low-dose naloxone 0.4 µg significantly accelerated motor block onset and reduced postoperative pain and pethidine requirements when added to 0.5% ropivacaine for ABPB in patients with ASA physical status I or II undergoing elective forearm and hand surgery. Second, all 120 blocks were clinically successful regardless of group assignment, confirming that sub-antagonist naloxone did not compromise block integrity. Third, low-dose naloxone 4 µg did not reduce pethidine requirement and was associated with a numerically higher proportion requiring pethidine than placebo (62.5% vs. 52.5%), although the difference was not statistically significant (P = 0.334). This opioid finding is distinct from the lower 30-minute mean VAS score in Group 2. The pethidine requirement pattern is hypothesis-generating and may suggest a dose-dependent shift in receptor modulation at higher sub-antagonist concentrations.

The mechanistic basis for these findings was originally established by Crain and Shen, who demonstrated that nanomolar concentrations of opioid antagonists selectively block Gs-coupled (excitatory) opioid receptor functions while preserving Gi-coupled (inhibitory) analgesic signaling (16, 17). As naloxone concentration increases beyond the ultra-low range, it progressively blocks both signaling pathways, potentially antagonizing rather than potentiating the analgesic properties of the coadministered local anesthetic. The numerically higher pethidine requirement in Group 2 than in Group 1, although not statistically significant, is consistent with this dose-dependent receptor-shift model. This observation warrants investigation in larger, adequately powered trials designed to detect differences between sub-antagonist doses.

The nonmonotonic pattern in postoperative pethidine requirement, with a reduction at 0.4 µg but no reduction and a numerically higher requirement at 4 µg, is consistent with the narrow therapeutic window hypothesis for sub-antagonist naloxone. At ultra-low concentrations, naloxone selectively antagonizes Gs-coupled excitatory opioid receptors, potentiating Gi-mediated analgesia; at higher concentrations, it begins to antagonize both pathways, potentially diminishing the net analgesic effect. This pattern has been observed in animal models but, to our knowledge, has not been directly demonstrated in an axillary block with ropivacaine (25). The co-primary outcomes, sensory and motor onset, were analyzed using a union-intersection approach, in which overall onset success was defined as a significant difference in at least one outcome. This is consistent with the finding that naloxone significantly accelerated motor, but not sensory, onset. The differential effect may reflect distinct pharmacological sensitivities of motor and sensory nerve fibers to the modulatory effects of ultra-low-dose naloxone and warrants further investigation.

The narrow therapeutic margin of perineural naloxone warrants emphasis. In this study, the 4 µg dose did not reduce pethidine requirement and was associated with a numerically higher 24-hour requirement (62.5%) than the saline control (52.5%), although this difference was not statistically significant. This trend suggests that exceeding the optimal ultra-low dose may shift the balance from selective Gi-coupled (inhibitory) opioid receptor potentiation toward nonselective antagonism of both Gs-coupled (excitatory) and Gi-coupled (inhibitory) pathways, thereby diminishing endogenous analgesia. Clinicians should be aware that the therapeutic window for perineural naloxone appears narrow, likely within the 0.1 - 0.4 µg range, and that doses as low as 4 µg may exceed this window and potentially antagonize endogenous analgesic mechanisms. This observation underscores the importance of precise dose selection in future trials and argues against arbitrary dose escalation in clinical practice.

Our opioid-sparing findings are consistent with prior evidence. Lee et al. reported that naloxone 100 ng added to ropivacaine prolonged femoral nerve block and reduced 24-hour opioid consumption (23). Amer and Omara demonstrated reduced analgesic requirements with naloxone 100 ng in intrapleural infiltration (26). Movafegh et al. reported significant ABPB prolongation with naloxone 100 ng added to lidocaine (20). Together with the present findings using 0.4 µg, these data suggest a narrow effective therapeutic window for peripheral nerve block adjuvant use, likely within the 0.1 - 0.4 µg range, and suggest that doses as low as 4 µg may exceed this window.

The absence of significant improvement in sensory block onset in this study differs from the block-duration findings reported in studies using bupivacaine. Marashi et al. reported prolonged sensory and motor block (21), whereas Al-Shukaili et al. reported prolonged sensory block but shortened motor block (22). These differences may reflect pharmacological differences between ropivacaine and bupivacaine or differences in the block approach. Ropivacaine has lower lipid solubility and a different pKa than bupivacaine, which may influence its interaction with sub-antagonist doses of naloxone at the receptor level.

Although mean VAS scores remained low during recovery (0.00 - 0.40), individual scores ranged from 0 to 4. The significant reduction in Group 3 at 30 minutes (P < 0.001), together with the reduction in the proportion requiring pethidine within 24 hours, supports the statistical and clinical significance of naloxone 0.4 µg. At 15 minutes, the overall ANOVA P value was 0.032 and the Group 1–Group 3 Tukey P value was 0.031; neither met the *Bonferroni*-adjusted threshold, and these findings require confirmation. Group 2 also had a lower mean VAS score than Group 1 at 30 minutes, with the same reported mean difference and confidence interval as Group 3. However, Group 2 did not have a lower pethidine requirement. The pain-score findings should therefore be distinguished from the dose-specific opioid-sparing effect.

Preblock fentanyl (1.5 µg/kg) was administered uniformly to all groups 10 minutes before block performance. This standardized premedication may have contributed to the low VAS scores across all groups; however, its uniform use makes between-group bias unlikely. The observed differences in 30-minute VAS scores and 24-hour pethidine requirement therefore reflect the incremental benefit of naloxone beyond the standardized systemic analgesic background.

Compared with established adjuvants, ultra-low-dose naloxone offers a favorable safety profile. Sehmbi et al. confirmed that dexmedetomidine and dexamethasone prolong peripheral nerve block but carry hemodynamic and steroid-related risks (12). Ultra-low-dose naloxone produced no hemodynamic instability, sedation, or adverse events in this study, and hemodynamic parameters remained stable throughout the intraoperative and recovery periods in all groups.

Several limitations warrant consideration. Block duration was not formally assessed as a prespecified outcome because of limitations in the original data collection protocol; this important gap should be addressed in future studies. Long-term outcomes beyond 24 hours were not evaluated. The single-center design and predominantly male population (83.3%) limit generalizability. The 3-minute assessment intervals may have been insufficient to detect subtle onset differences. The baseline DBP imbalance despite randomization is an acknowledged confounder. Conclusions about receptor-level mechanisms are speculative because no direct receptor measurements were performed. Multicenter trials with stratified randomization, formal assessment of block duration, finer assessment intervals, and larger sample sizes are needed to confirm and extend these findings (25).

These findings should be considered preliminary and should not be generalized beyond the population studied, namely patients with ASA physical status I or II undergoing forearm and hand surgery with ABPB using 0.5% ropivacaine, without further investigation.

### 5.1. Conclusions

In patients with ASA physical status I or II undergoing elective forearm and hand surgery, adding ultra-low-dose naloxone 0.4 µg to 0.5% ropivacaine for ultrasound-guided ABPB significantly accelerated motor block onset and reduced the proportion requiring pethidine within 24 hours in this single-center study. These findings support consideration of naloxone 0.4 µg as a safe adjuvant in this specific setting. Low-dose naloxone 4 µg was associated with a lower mean VAS score at 30 minutes but did not reduce pethidine requirement; the proportion requiring pethidine was numerically higher than with placebo, although the difference was not statistically significant. This is a hypothesis-generating observation consistent with the biphasic dose-response model of sub-antagonist opioid receptor modulation. Given the single-center design, limited sample size, lack of formal block duration assessment, and 24-hour follow-up, the results should be considered preliminary and require confirmation in larger, adequately powered multicenter trials. The therapeutic window for naloxone as an adjuvant appears narrow; clinicians should favor the 0.4 µg dose, as higher sub-antagonist doses such as 4 µg may diminish rather than enhance endogenous analgesia. Given the trend toward an increased pethidine requirement in the 4 µg group, we strongly caution against exceeding the ultra-low-dose range (≤ 0.4 µg) in clinical practice, as higher sub-antagonist doses may antagonize endogenous analgesic mechanisms. Future studies should precisely define the upper limit of the therapeutic window for perineural naloxone.

## Data Availability

Data are available upon reasonable request from the corresponding author.

## Clinical Trial Registration Code:

IRCT20170105031787N6.
